# Patient Preferences for Lung Cancer Treatments: A Study Protocol for a Preference Survey Using Discrete Choice Experiment and Swing Weighting

**DOI:** 10.3389/fmed.2021.689114

**Published:** 2021-08-02

**Authors:** Dario Monzani, Serena Petrocchi, Serena Oliveri, Jorien Veldwijk, Rosanne Janssens, Luca Bailo, Meredith Y. Smith, Ian Smith, Elise Schoefs, Kristiaan Nackaerts, Marie Vandevelde, Evelyne Louis, Herbert Decaluwé, Paul De Leyn, Hanne Declerck, Eva G. Katz, Francesco Petrella, Monica Casiraghi, Ilaria Durosini, Giulia Galli, Marina Chiara Garassino, G. Ardine de Wit, Gabriella Pravettoni, Isabelle Huys

**Affiliations:** ^1^Applied Research Division for Cognitive and Psychological Science, IEO, European Institute of Oncology IRCCS, Milan, Italy; ^2^Department of Oncology and Hemato-Oncology, University of Milan, Milan, Italy; ^3^Erasmus Choice Modelling Centre, Erasmus University Rotterdam, Rotterdam, Netherlands; ^4^Erasmus School of Health Policy & Management, Erasmus University Rotterdam, Rotterdam, Netherlands; ^5^Julius Center for Health Sciences and Primary Care, University Medical Center Utrecht, Utrecht University, Utrecht, Netherlands; ^6^Department of Pharmaceutical and Pharmacological Sciences, KU Leuven, Leuven, Belgium; ^7^Alexion Pharmaceuticals, Inc., Boston, MA, United States; ^8^University of Southern California School of Pharmacy, Los Angeles, CA, United States; ^9^Department of Respiratory Oncology, University Hospital Leuven, Leuven, Belgium; ^10^Department of Thoracic Surgery, KU Leuven, Leuven, Belgium; ^11^Janssen Research and Development, LLC, Raritan, NJ, United States; ^12^Thoracic Surgery Division, IEO, European Institute of Oncology IRCCS, Milan, Italy; ^13^Department of Medical Oncology, Fondazione IRCCS Istituto Nazionale dei Tumori, Milan, Italy; ^14^University of Chicago Department of Medicine Section Hematology/Oncology, Chicago, IL, United States

**Keywords:** non-small cell lung cancer, patient preference, discrete choice experiment, swing weighting, educational tool, health literacy, health locus of control, quality of life

## Abstract

**Background:** Advanced treatment options for non-small cell lung cancer (NSCLC) consist of immunotherapy, chemotherapy, or a combination of both. Decisions surrounding NSCLC can be considered as preference-sensitive because multiple treatments exist that vary in terms of mode of administration, treatment schedules, and benefit–risk profiles. As part of the IMI PREFER project, we developed a protocol for an online preference survey for NSCLC patients exploring differences in preferences according to patient characteristics (preference heterogeneity). Moreover, this study will evaluate and compare the use of two different preference elicitation methods, the discrete choice experiment (DCE) and the swing weighting (SW) task. Finally, the study explores how demographic (i.e., age, gender, and educational level) and clinical (i.e., cancer stage and line of treatment) information, health literacy, health locus of control, and quality of life may influence or explain patient preferences and the usefulness of a digital interactive tool in providing information on preference elicitation tasks according to patients.

**Methods:** An online survey will be implemented with the aim to recruit 510 NSCLC patients in Belgium and Italy. Participants will be randomized 50:50 to first receive either the DCE or the SW. The survey will also collect information on participants' disease-related status, health locus of control, health literacy, quality of life, and perception of the educational tool.

**Discussion:** This protocol outlines methodological and practical steps to quantitatively elicit and study patient preferences for NSCLC treatment alternatives. Results from this study will increase the understanding of which treatment aspects are most valued by NSCLC patients to inform decision-making in drug development, regulatory approval, and reimbursement. Methodologically, the comparison between the DCE and the SW task will be valuable to gain information on how these preference methods perform against each other in eliciting patient preferences. Overall, this protocol may assist researchers, drug developers, and decision-makers in designing quantitative patient preferences into decision-making along the medical product life cycle.

## Introduction

Lung cancer (LC) is the most prevalent and deadliest cancer worldwide with at least 85% of them affected by non-small cell lung cancer (NSCLC) (1). Late diagnosis is one of the fundamental reasons for the high number of deaths, with more than 70% of new cases detected too late for trying curative treatments (2). Moreover, NSCLC patients are a vulnerable population both because most patients with a diagnosis of LC are 65 or older and because they reported high levels of physical and psychological distress and suffering (3).

In the last few years, patient preference (PP) for treatment outcomes has been receiving increasing attention as a relevant source of complexity (4–6). Specifically, PP heterogeneity arises when patients differ in how they value specific treatment attributes or outcomes. Treatment options for NSCLC may vary widely according to disease stage. Traditionally, treatment options for advanced-stage NSCLC have consisted of chemotherapy, radiation therapy, or a combination, whereas surgery is a more feasible treatment option for the early stage. However, the current treatment paradigm for advanced-stage NSCLC has shifted due to the development and introduction of drug therapies with novel mechanisms of actions. In particular, immunotherapy with immune checkpoint inhibitors (ICIs) has been developed (7, 8).

Nevertheless, without clinical trials comparing different NSCLC treatment options, there is no clinical evidence that allows to determine a clinically superior treatment for advanced stage patients. Further, NSCLC treatments (including immunotherapy and chemo-immunotherapy) have different treatment characteristics that may influence treatment decisions. NSCLC treatments vary in terms of mode of administration, treatment schedules, and benefit–risk profiles. For instance, while chemo-immunotherapy is administered as an intravenous infusion lasting at least 4–5 h, immunotherapy has a much shorter infusion time of about 1 h. Toxicity profiles also differ between NSCLC treatments. For example, chemotherapy can carry both acute and late toxicities; while the former includes nausea, vomiting, paresthesia, anemia, fatigue, and fever, the latter can include persistent neurotoxicity and infertility. However, immunotherapy is typically better tolerated, as the most common side effects are rash, itch, mild diarrhea, fatigue, and subclinical thyroid dysfunction. Therefore, in view of the existence of multiple treatments with varying modes of administration, treatment schedules, benefit–risk profiles, no clinical superior treatment, and treatment options that vary widely according to disease stage and severity (9), PP may play a pivotal role in understanding the relative value of different therapeutic options and their attributes for NSCLC patients. Decisions concerning NSCLC treatment can be considered a PP-sensitive decision, and a better understanding of the patient's experience with NSCLC is therefore pivotal in these circumstances. For example, NSCLC patients have a low life expectancy that varies according to the disease stage (10), and the understanding of their preference regarding treatment options, the maximum acceptable risk (MAR), and the minimum acceptable benefit (MAB) they would be willing to accept for treatment alternatives may inform stakeholders.

A recent systematic review suggested that, alongside socio-demographic (e.g., age) and clinical characteristics (e.g., disease stage), patients' psychological aspects (e.g., health literacy and health locus of control) may have an impact on PP and health-related decisions (11). However, since no empirical evidence is reported on how these psychological aspects may affect preferences of LC patients for their treatments, based on the perspective of personalized medicine (12–14), this information would be essential to propose and deliver personalized treatments for NSCLC.

PP can be elicited through various quantitative techniques. DCE is the most common method for eliciting PP in the life cycle of medical products (15). It is a quantitative method to assess PP by asking respondents to state their choice over sets of hypothetical alternatives defined by different levels of several characteristics, known as attributes. These responses are then considered to infer the value placed on each attribute. These responses are then considered to estimate the strength of preference for change in levels of considered characteristics (16). Another quantitative preference method that has gained attention is swing weighting (SW), which has emerged during the last years as an alternative method for eliciting PP (17). SW asks respondents to indicate which attribute they would prioritize to improve from the worst to the best.

SW and DCE are two widely used PP methods in the field of health. Notwithstanding, research directly comparing DCE and SW is particularly lacking (18, 19). Methods such as SW that do not force patients' simultaneous trade-offs between multiple attributes (20) are considered simpler. Otherwise, others suggested that direct comparisons in a DCE can be easier for patients (18). Therefore, the aim of this study is to compare the performance and results of DCE and SW in a common preference context through empirical research.

Even if there are conceptual, methodological, and practical differences between DCE and SW, there is limited empirical evidence on how the SW and DCE perform against each other in eliciting PP. From a methodological stance, it would therefore be relevant to evaluate and compare these two different methods in eliciting PP for NSCLC treatments.

### Study Objectives

This study is part of the Innovative Medicines Initiative (IMI) Patient Preferences in Benefit-Risk Assessments during the Medical Product Lifecycle (PREFER) project (21). This 5-year research project will develop evidence-based recommendations to guide industry, regulatory authorities, and health technology assessment bodies on when and how PP on benefits and harms should be assessed and used to inform decision making throughout the medical product life cycle (for a brief overview, see https://www.imi-prefer.eu/ or https://www.imi.europa.eu/projects-results/project-factsheets/prefer).

This study has both clinical and methodological endpoints. The clinical endpoints of this study are:

- What are the MAR and MAB that patients would accept for treatment alternatives?
- What are the clinical, demographic and psychological variables that may explain patients' preferences? In particular, we will explore to what extent PPs vary with disease stage and line of treatment, age, gender, educational level, and psychological characteristics of the patients (i.e., health literacy and health locus of control).

As methodological endpoints, the study will assess the following:

- How similar are the results acquired from the two different methods (i.e., DCE and SW) applying the same set of attributes on the same population? To what extent assessing preferences for treatment alternatives in NSCLC patients with a DCE will provide comparable information if compared to a different method like the SW? The aim is to highlight the strengths and weaknesses of two different methodologies in identifying and profiling different treatments within the context of patient management.
- Would an educational tool be perceived as being a useful way to provide information on treatment attributes and attribute levels, and to teach participants how to perform different choice tasks?

## Methods

### Design

This study will be implemented following the International Society for Pharmacoeconomics and Outcomes Research (ISPOR; 22) guidelines for eliciting preferences and conducting PP studies. It is a cross-sectional study consisting of an online survey administered to NSCLC patients in Belgium and Italy. The NSCLC participants will be randomized 50:50 to first receive either a DCE or SW task. This is to avoid order effects that could introduce confounding. Before completing the DCE and SW tasks, patients will receive a description of the attributes included in the two choice tasks and instructions about how to complete each task using an educational tool.

### Procedure

Prior to completing the survey, eligible NSCLC participants will be provided with a Participant Information Sheet containing the goals of the study, expectations for participation, and contact information of the study team by a nurse, a clinician, or a researcher. Eligible participants will receive a personalized link to the online survey. Only participants who provide their informed consent as part of the survey will be able to complete the online survey. The survey will be administered by Sawtooth software v.9.9.2 offered *via* a server of Uppsala University.

A first version of the survey will be pretested in a small group of five patients by means of think-aloud interviews. These patients will provide feedback on the survey to make sure that it is understandable and is not too time consuming or burdensome. The tool content and format will be revised in response to this feedback.

The study will be conducted in compliance with the EU General Data Protection Regulation (GDPR) and all other European and national legislations. Additionally, this study was approved by the Ethical Committee of the European Institute of Oncology IRCCS (IEO, Milan, Italy; reference R1142/20-IEO 1206) and the “Ethische Commissie Onderzoek UZ/KU Leuven” (Belgium; reference S64022).

### Participants

**Figure 1:**
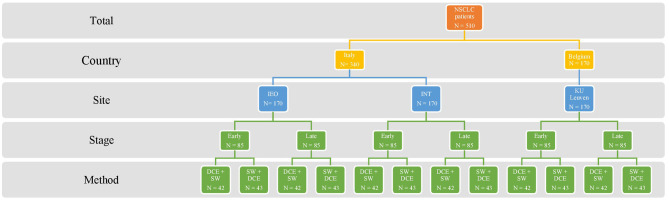
Targeted sample distribution. NSCLC, non-small cell lung cancer; IEO, European Institute of Oncology; INT, National Institute of Cancer; KU Leuven, KU Leuven University Hospital in Leuven; Early, early stages (I–II); Late, late stages (III–IV); DCE, discrete choice experiment; SW, swing weighting.

The study aims to recruit 510 NSCLC patients from three cancer treatment centers (*N* = 170 per center) in Italy (*N* = 340) and Belgium (*N* = 170). Italian patients will be recruited at the European Institute of Oncology and the National Institute of Cancer in Milan. Belgian patients will be recruited at the Respiratory Oncology Department and the Department of Thoracic Surgery of the KU Leuven University Hospital in Leuven. Each cancer center treats approximately 500 NSCLC patients yearly. NSCLC patients in different stages (stages I–IV) will be recruited and divided into two groups (see Figure 1): 255 early stages (stages I and II) and 255 later stages (stages III and IV).

Treatment options for patients, their prognosis, and their perception of the disease widely differ according to the stage of LC. The treatment attributes included in the study could be more familiar or realistic for late-stage LC patients at the time of their participation. However, we include both early- and late-stage patients since the hypothetical scenarios presented would be relevant to both groups; some of the early-stage patients could, in the future, be confronted with the same therapy choices as patients in later stages. In addition, one of the aims of this study is to investigate whether disease stage, alongside other clinical variables (i.e., line of treatment), socio-demographic (i.e., age, gender, and educational level), and psychological characteristics of the patients (i.e., health literacy and health locus of control), would be related to PP heterogeneity. Table 1 shows participants' inclusion and exclusion criteria.

**Table 1:** Inclusion and exclusion criteria.

**Inclusion criteria**	**Exclusion criteria**
NSCLC patients	Inability to consent or assent by themselves
I–IV stages of lung cancer	Aged < 18
Aged ≥ 18	Inability to read, speak, and understand either Italian or Dutch
Able to give informed consent	
Able to read, speak, and understand either Italian or Dutch	

### Sample Size Estimation

For SW, no specific sample size is required since the sample size does not depend on the magnitudes of the utility differences (22). Therefore, the sample sizes will be dictated by requirements for the DCE. To the best of our knowledge, there are no statistical tools able to estimate the sample size for a DCE. Therefore, we referred to the literature to estimate the sample size for the present research. Most published choice experiments have a sample size of between 100 to 300 respondents (23). However, according to Bekker-Grob et al. (24), the minimum sample size depends on several criteria, including the question format, the complexity of the choice task, the desired precision of the results, and the need to conduct subgroup analyses (25, 26). Given this, we have determined that, based on the sample size requirements for both methods and taking into account the number of research questions this study anticipates to answer, a sample size of 510 respondents (170 per site) is expected to provide enough information to identify preferences in each country and comparisons across countries and disease groups with acceptable precision.

### Measures and Development of Preference Elicitation Tasks

**Figure 2:**
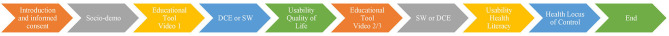
Order of the questions in the survey. DCE, discrete choice experiment; SW, swing weighting.

The order of the questions in the online survey is shown in Figure 2.

Socio-demo questions on gender, age, education, relationship status, history of LC in the family, age of the diagnosis of LC, types and number of lines of treatments received, participation in clinical trials, contact with other patients, or patient organizations will be asked. Patients' health-related quality of life will be assessed using the five-level EQ-5D version [EQ-5D-5L; (27)] which comprises five dimensions: mobility, self-care, usual activities, pain/discomfort, and anxiety/depression. The measurement of health-related quality of life and socio-demographic and clinical variables will allow a more precise characterization of patients.

#### Attributes and Levels Development

The selection of the attributes and levels of the DCE and the SW arise from a previous qualitative study (28, 29). Through focus-group discussions conducted in Belgium and in Italy, patients highlighted themes reflecting positive effects, or expected gains of treatment, and negative effects or adverse events that negatively impacted their daily functioning. Twenty-one themes emerged from those discussions, mainly consistent among patients from Belgium and Italy (29).

Two additional focus groups, one in Belgium and one in Italy, were conducted with 13 NSCLC stage III and IV patients (age range 48–70: *M* = 58, *SD* = 7.1; 54% men; *N* = 7 in Belgium; *N*= 6 in Italy). Results were used to derive attributes and attribute levels. From the 21 themes that emerged during the first round of discussions with patients (29), participants selected and rank-ordered the most important ones. The four most important positive characteristics were greater life expectancy, decrease in cancer growth, cancer remission, and the maintenance of daily functioning, and eight most important negative characteristics were severity of skin problems, nausea, serious infections, hair loss, and infusion reactions, gravity of fatigue, probability of renal failure, and probability of cognitive limitation.

Multi-stakeholder discussions involving clinicians and preference research experts were then organized to further discuss the characteristics and define the final attributes, their associated levels, and their explanations. For parsimony and to avoid overlapping categories, the three categories greater life expectancy, decrease in cancer growth, and remission were combined into “chance to be alive at least 5 years after the initiation of the therapy.” The category of maintenance of daily functioning was renamed as “chance to carry out activities of daily living” (e.g., social interaction, working productivity, household activities, practice sports, being able to go on holiday). Therefore, the list that was evaluated by three clinicians (two in Italy and one in Belgium) comprised two positive characteristics (i.e., the probability of >5 years of survival and chance to carry out activities of daily living) and nine negative characteristics (i.e., severity of skin problems, nausea, serious infections, hair loss, and infusion reactions, gravity of fatigue, probability of renal failure, and probability of cognitive limitation).

The multi-stakeholder discussions resulted in the deletion of four characteristics: the severity of infections, severity of infusion reactions, probability of cognitive limitations, and probability of renal failure. All those characteristics were deleted because according to the clinicians they are deemed as rare in NCSLC. The severity of the nausea was deleted because it can be treated and controlled by other existing drugs. Since attributes should be unambiguous, clear, tradable, and distinctly different from other included attributes (30), the chance to carry out activities of daily living was deleted from the list because (a) it is difficult to find a univocal definition for that feature, (b) it overlaps with the other included attributes (it is an overarching theme encompassing the other, more specific, attributes), and (c) it may be interpreted differently by different patients. The attribute of treatment modality (i.e., how the treatment is given to you) was added because it differs across different treatments that have been developed for NSCLC and knowing whether this attribute actually influences patients' decisions could be valuable information in HTA and regulatory decisions. The final list is composed of five attributes with three levels each (see Table 2). The same attributes and levels have been implemented in both DCE and SW to allow for their comparison.

**Table 2:** Attributes, definitions, and levels.

**Attribute**	**Definition**	**Levels**
How the treatment is given to you	How the cancer treatment is given to you and the length of time each treatment takes.	This can either be: - Infusion (injection administered into your veins) that requires a hospital stay of 1 day (about 24 h) - Infusion (injection administered into your veins) that requires a hospital stay of half a day (about 12 h) - Oral treatment (by swallowing), and no hospital stay is required
Chance of surviving 5 years after starting this cancer treatment	The chance of still being alive 5 years after starting this cancer treatment	This chance can either be: - 10%—meaning that 10 people out of 100 people that started the treatment are still alive after 5 years, and 90 people died within those 5 years - 20%—meaning that 20 people out of 100 people that started the treatment are still alive after 5 years, and 80 people died within those 5 years - 40%—meaning that 40 people out of 100 people that started the treatment are still alive after 5 years, and 60 people died within those 5 years
Chance of long-lasting skin problems	The chance that skin problems occur after treatment. This skin problem lasts at least a month and could be a rash, severe itching, bleeding, and/or dryness	This chance can either be: - 10% (10 out of 100)^a^ - 20% (20 out of 100)^a^ - 40% (40 out of 100)^a^
Chance of being extremely tired	This refers to feeling completely exhausted and lacking energy even after limited activities. It lasts as long as the treatment takes to be administered.	This chance can either be: - 10% (10 out of 100)^a^ - 40% (40 out of 100)^a^ - 60% or (60 out of 100)^a^
Severity of hair loss	The type and amount of hair loss. It lasts as long as the treatment takes to be administered.	This can either be: - No hair loss - Weakening/thinning of hair - Complete loss of hair

a *The percentage is presented also in a graphical form displaying 100 stylized human figures and highlighting a number of them with a color corresponding to the given percentage*.

#### DCE and SW Task Construction

The DCE (15) is a quantitative method to assess PP by asking respondents to state their choice over sets of hypothetical alternatives defined by combinations of different levels of several characteristics, hence the attributes. In the present research, respondents will be presented 12 choice tasks, following ISPOR guidelines. Each choice task consists of selecting between two alternative options which describe a hypothetical treatment A and treatment B (see Table 3 for an example of choice task). In each treatment, the five established attributes are linked to one of the three possible levels.

**Table 3:** Example of a DCE choice task created for the study.

	**Treatment A**	**Treatment B**
How the treatment is given to you	Intravenous infusion lasting 24 h	Intravenous infusion lasting 12 h
Chance of survival 5 years after starting this cancer treatment	10% (10 out of 100)	10% (10 out of 100)
Chance of long-lasting skin problems	40% (40 out of 100)	10% (10 out of 100)
Chance of being extremely tired	60% (60 out of 100)	10% (10 out of 100)
Severity of hair loss	Weakening/thinning	No hair loss

NGene 1.2.1 software (31) will be used to create a Bayesian D-efficient design. Prior information used to generate this design was based on the previous literature and best guesses for the pilot study, and outcomes of initial analysis (conditional logit) of pilot data for the main survey. Dominant alternatives where one alternative is clearly “better” than the other alternative were excluded from the design. For both the pilot and the final design, a total of 36 unique choice tasks were generated, which were divided over three blocks; each choice task was assigned to only one of the blocks (each patient answered only 12 choice tasks). Patients will be randomized to either one of the blocks. Interactions between 5-year survival and risk or extreme tiredness, 5-year survival and risk of skin problems, and 5-year survival and mode of administration were included in the design.

The SW (17) is a method in which respondents are asked to indicate which attribute, among a provided list, they want to improve (i.e., to “swing”) from the worst to the best level first. After making this choice, respondents are asked to indicate which is the second attribute they want to improve from worst to the best. This is repeated until all attributes are ranked. Afterward, a drag-and-drop method allows patients to further adjust their selection after the ranking is completed. The order in which the attributes will be presented in the description will be randomized. Table 4 provides an example of a scenario with attributes and attribute levels.

**Table 4:** Example of the SW choice task—attribute ranking.

	Drag the most important improvement here
**Severity of hair loss**	
Total hair loss → no hair loss	
**Chance of being extremely tired**	
60% (60 out of 100) → 10% (10 out of 100)	
**Chance of survival 5 years after starting this cancer treatment**	
10% (10 out of 100) → 40% (40 out of 100)	
**Chance of long-lasting skin problems**	
40% (40 out of 100) → 10% (10 out of 100)	
**How the treatment is given to you**	
Intravenous infusion lasting 24 h → oral treatment (pill swallowing)	
	Drag the least important improvement here

Participants will then be asked to assign points to each attribute they have ranked in the previous step. The first attribute automatically receives 100 points; participants have to assign points to all the other attributes (i.e., “weighting”). See Table 5.

**Table 5:** Example of the SW choice task—point allocation.

The improvement of the following characteristic was found to be the most important for you:
**Chance of survival 5 years after starting this cancer treatment**
For this reason, we assign it a score of 100 points.
Please assign a score to the second most important improvement by moving the cursor on the bar below, so that it reflects its importance vs. the first.The second improvement was:
**How the treatment is given to you**
** 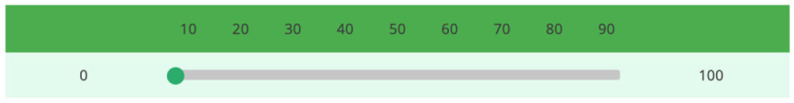 **
We ask you to do the same with all the improvements shown below.Warning! We remind you that if the first improvement is 100 points worth and you have assigned for example 50 points to the second improvement, to the third improvement you will have to assign a score lower than 50 points and so on for the subsequent ones classified by you as less important. Remember that you can go back and edit your answers at any time.
**Chance of being extremely tired**
** 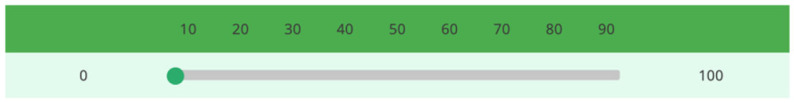 **
**Chance of long-lasting skin problems**
** 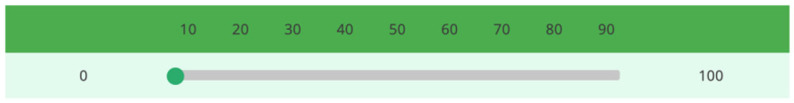 **
**Severity of hair loss**
** 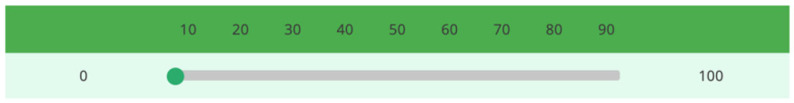 **

#### Education Tool

An educational tool will provide study participants with information regarding the related attributes, levels, and survey technique. The tool leverages digital technology to educate patients on the attributes and attribute levels and instruct them in completing the choice tasks associated with the two types of preference elicitation methods (i.e., SW and DCE) included in the study. The content and format of the educational tool adhere to plain language principles, are written at an intermediate reading level, include visualizations to demonstrate key learning points, and enable patients to practice completing several choice tasks using a fictitious medication (a cold medicine) as an example. Three videos will be created *ad hoc* with colored graphical animations accompanied by a descriptive voiceover. One video explains to the participants the five attributes and their levels. A second video describes the functioning of the DCE and a third video illustrates the functioning of the SW.

#### Psychological Measures

Health literacy refers to patients' ability to read, understand, and use healthcare information appropriately and the ability to apply and manipulate numerical concepts (32, 33). This construct has been conceptualized in terms of subjective health literacy or objective evaluations. In the present research, health literacy will be measured with the Chew's Set of Brief Screening Questions (32) and the Newest Vital Sign (33). The Chew's Set of Brief Screening Questions is a self-reported, validated subjective measure of health literacy containing three items. The Newest Vital Sign is an objective and validated measure of health literacy in which patients are given an ice cream nutrition label containing health-related information and they have to answer six questions about this nutrition label.

A health locus of control is defined as a generalized expectation about whether one's health is controlled by one's own behavior or by external forces (34). An individual with a high internal locus of control believes that outcomes are a direct result of his or her own behavior. In contrast, an individual with a high external locus of control believes that outcomes are a result of either chance or powerful other people, such as physicians. The health locus of control will be measured with the Multidimensional Health Locus of Control Scale—Form C (34), an 18-item, general-purpose, condition-specific locus of control scale that can easily be adapted for use with any medical or health-related condition.

### Statistical Analyses

The clinical objective of this study is to assess patients' preferences for relevant treatment attributes related to LC treatment alternatives. To determine preferences of study participants, attribute (level) estimates and the conditional relative importance of attributes will be explored in the DCE using conditional logit models. For the final analyses, preferences for attributes of NSCLC treatment random parameters logit (RPL) modeling and latent class analysis (LCA) will be considered. Final decisions on the modeling procedure will be made once data collection has been completed and explored. This decision will be based on model fit and clinical interpretation. Different models might be used to answer the different research questions in this case study. Heterogeneity of preferences and the impact of participant characteristics among which psychological variables will be investigated *via* LCA or subgroup analyses using RPL.

For the SW, each attribute is first ranked by the participants, and then the relative importance is indicated through point allocation. The rank order will be turned into weights using a rank-to-weight method, rank order centroid method (35). Using this approach, the average weight per attribute over the entire group of respondents is then calculated and it becomes possible to statistically investigate whether weights differ between subgroups.

The MAR and MAB values for benefits and risks of interest will be determined based on DCE outcomes.

The methodological objective of this study is to evaluate the similarity of DCE and SW in assessing preferences for treatment alternatives in NSCLC. The results of the DCE and SW will be compared qualitatively and quantitatively to evaluate whether the results are different when applying two different methods. As a first step, validity checks, completion time (through log information), dropout, and response to feedback questions will be compared between the two methods. Second, the relative importance of the included attributes will be compared between the two methods.

PP heterogeneity and the influence of participants' clinical (i.e., disease stage and line of treatment), socio-demographic (i.e., age, gender, and educational level), and psychological characteristics (i.e., health literacy and health locus of control) will be investigated by applying appropriate statistical models including LCA and/or subgroup analyses.

Finally, the evaluation of patients' perceived usefulness of the educational tool will be assessed by conducting descriptive analysis on patients' rating of efficacy and usefulness, as well as correlational analyses assessing associations between this rating and patients' health literacy and numeracy, and clinical and sociodemographic variables.

### Handling Missing Data

Respondents who choose not to answer certain demographic or treatment history questions will be noted in the summary statistics, and their responses will be included in the DCE; however, if data required to assign an individual to a subgroup are missing, that individual will be excluded from the subgroup analysis (pairwise deletion). Respondents who do not answer at least one DCE question and at least one SW question in the survey, or who always select the same alternative (either treatment A or treatment B) in all of the DCE questions (“flatliners”), will be analyzed separately as a part of the quality assessment evaluation. For comparing DCE to SW, we will include respondents who fully completed both methods, whereas for the separate analyses on the DCE or SW, we will include respondents that completed parts of the methods.

### Dissemination

The findings of this study will be disseminated *via* international peer-reviewed journals and scientific conferences. The study protocol has been registered on Health Preference Study and Technology Registry (ID: #190183-001). A summary of the study results will also be written for the lay audience and made available to participants and relevant patient organizations for distribution on their own channels. Patient organizations will be approached to help to disseminate the study results to their members.

## Discussion

Decisions related to LC treatments based on their characteristics are preference-sensitive decisions. Specifically, available therapeutic options for these patients have different treatment characteristics and likely impact PP and influence subsequent decisions. For instance, patients with NSCLC might be asked to choose between an aggressive therapeutic option with possible detrimental effect on their quality of life and an alternative treatment with less effectiveness but fewer harmful effects on quality of life. Therefore, it is essential to understand PP, which attributes patients perceive as more relevant, and the amount of risk they may be willing to tolerate in exchange for a minimal level of benefit.

This quantitative protocol describes a study aiming to identify patient-relevant LC treatment attributes and to understand which treatment characteristics are most important for advanced LC patients. The present study has several methodological and clinical endpoints. One main methodological endpoint is to ascertain how much the results acquired from the two methods, DCE and SW, would be similar when evaluated on a sample of NSCLC patients. There is no consensus on which is the best method to gather quantitative treatment preference data with multiple options ranging from simple ranking exercises to complex trade-off methods currently employed. Thus, this study aims to evaluate to what extent assessing preferences for treatment alternatives in NSCLC with a more demanding instrument like DCE will provide higher-quality information if compared to a less expensive method like the SW task. This will inform further research on PP and contribute to the elaboration of international recommendations developed by the PREFER initiative on how preference data should be measured and included into the treatment development life cycle.

Another methodological endpoint is the evaluation of whether an educational tool would be perceived as being useful in providing information on treatment attributes and attribute levels, and to teach participants how to perform different choice tasks. Results from this study may inform further research on the application of video in supporting fragile patients during survey completion.

The clinical endpoints evaluate the MAR and MAB that patients would accept for treatment alternatives and what are the clinical, demographic, and psychological variables that may explain heterogeneity in patients' preferences. The understanding of what patients perceive as important treatment attributes and the amount of risk they may be willing to tolerate in exchange for a minimal level of benefit may inform preference-sensitive decision-making. Moreover, in line with the objectives of the PREFER initiative, the assessment of what individual psychological characteristics may explain preference heterogeneity can enrich the knowledge on PP and guide future research. Including patients that are currently in different stages of the NSCLC disease, this study will allow us to consider the stage of their disease as a factor that might potentially explain differences in their preferences that emerge as relevant to their treatment decision-making.

This newly acquired knowledge will be useful to enhance medical decision-making, promote personalized treatment decisions, and propose and deliver tailored treatments for NSCLC. Overall, the results coming from this quantitative study will be especially relevant to better understand PPs for different NSCLC treatment attributes, inform decision-making regarding available NSCLC therapeutic options, and promote patient-centered healthcare in the development, evaluation, and medical treatment of LC.

## Data Availability Statement

The original contributions generated for the study are included in the article/supplementary material, further inquiries can be directed to the corresponding author/s.

## Ethics Statement

The study was approved by the Ethical Committee of the European Institute of Oncology IRCCS (IEO, Milan, Italy; reference R1142/20-IEO 1206) and the Ethische Commissie Onderzoek UZ/KU Leuven (Belgium; reference S64022). Participants signed a consent form before participating.

## Author Contributions

DM, JV, RJ, LB, MYS, IS, EGK, ID, AdW, GP, and IH: initial conceptualization and design of the study. SP, SO, KN, MV, EL, HDeca, PD, HDecl, ES, GG, MG, MC, and FP: further adaptation and improvement of the design of the study. DM and SP: drafted the manuscript. All the authors provided critical revision of the manuscript, and have read and approved the final manuscript.

## Conflict of Interest

MYS is employed at Alexion Pharmaceuticals Inc., and EGK is employed at Janssen Research and Development. The remaining authors declare that the research was conducted in the absence of any commercial or financial relationships that could be construed as a potential conflict of interest.

## Publisher's Note

All claims expressed in this article are solely those of the authors and do not necessarily represent those of their affiliated organizations, or those of the publisher, the editors and the reviewers. Any product that may be evaluated in this article, or claim that may be made by its manufacturer, is not guaranteed or endorsed by the publisher.
